# Overexpression of p49/STRAP alters cellular cytoskeletal structure and gross anatomy in mice

**DOI:** 10.1186/1471-2121-15-32

**Published:** 2014-09-02

**Authors:** Xiaomin Zhang, Gohar Azhar, Steven C Rogers, Stephen R Foster, Shaoke Luo, Jeanne Y Wei

**Affiliations:** 1 Reynolds Institute on Aging & Department of Geriatrics, University of Arkansas for Medical Sciences, 4301 West Markham St. #748, Little Rock, AR 72205, USA; 2 Geriatric Research Education and Clinical Center, Central Arkansas Veterans Healthcare System, Little Rock, AR 72205, USA

**Keywords:** Transcription cofactor, Homology, Subcellular localization, Cell morphology, Transgenic mice, CMV promoter reactivation

## Abstract

**Background:**

The protein p49/STRAP (SRFBP1) is a transcription cofactor of serum response factor (SRF) which regulates cytoskeletal and muscle-specific genes.

**Results:**

Two conserved domains were found in the p49/STRAP protein. The SRF-binding domain was at its N-terminus and was highly conserved among mammalian species, xenopus and zebrafish. A BUD22 domain was found at its C-terminus in three sequence databases. The BUD22 domain was conserved among mammalian p49/STRAP proteins, and yeast cellular morphogenesis proteins, which is involved in ribosome biogenesis that affects growth rate and cell size. The endogenous p49/SRAP protein was localized mainly in the nucleus but also widely distributed in the cytoplasm, and was in close proximity to the actin. Transfected GFP-p49/STRAP protein co-localized with nucleolin within the nucleolus. Overexpression of p49/STRAP reduced actin content in cultured cells and resulted in smaller cell size versus control cells. Increased expression of p49/STRAP in transgenic mice resulted in newborns with malformations, which included asymmetric abdominal and thoracic cavities, and substantial changes in cardiac morphology. p49/STRAP altered the expression of certain muscle-specific genes, including that of the SRF gene, which is a key regulator of cardiac genes at the developmental, structural and maintenance level and has two SRE binding sites.

**Conclusions:**

Since p49/STRAP is a co-factor of SRF, our data suggest that p49/STRAP likely regulates cell size and morphology through SRF target genes. The function of its BUD22 domain warrants further investigation. The observed increase in p49/STRAP expression during cellular aging may contribute to observed morphological changes in senescence.

## Background

The protein p49/STRAP was identified in our laboratory in search of proteins that regulate cytoskeletal genes related to cardiac function [1]. Our initial observations indicated that p49/STRAP is a cofactor of serum response factor (SRF), contributing to the regulation of SRF target genes. This gene was later named by The Human Genome Organization (HUGO) as SRFBP1, based on our initial finding that p49/STRAP binds to SRF.

Serum response factor is an important transcription factor that regulates a number of immediate-early genes, cytoskeletal and muscle-specific genes. SRF has been reported to exhibit functional interactions with a number of SRF cofactors and/or binding proteins in the regulation of SRF target genes [2-6]. These interactions likely modulate SRF function and may also enable SRF to mediate tissue-specific regulation of its target genes at different developmental stages [6,7]. A model of transcriptional regulation by SRF and its binding proteins has been proposed, in which the interaction of multiple proteins (including p49/STRAP) affects the outcome of the expression of SRF target genes [1]. SRF has two functional domains. The N-terminus is a DNA binding domain that binds to the serum response element or CArG box and the C-terminus is a transactivation domain [8]. The p49/STRAP protein interacts directly with the SRF transcriptional activation domain. Therefore, the level of p49/STRAP expression and its affinity to SRF protein may have a significant impact on SRF activation [1].

The p49/STRAP mRNA and protein are expressed in multiple tissues, including heart, brain, liver, skeletal muscle and kidney [1]. An increase of p49/STRAP expression has been observed in the myocardium of adult human and mouse with advancing age, and also in transgenic mouse models of cardiomyopathy [1,9]. However, the functional domains of the p49/STRAP protein and the effect of increased in-vitro and in-vivo p49/STRAP expression remain to be defined. In the present study, the p49/STRAP protein sequence was analyzed for conserved domains. A SRF-binding domain was found at the N-terminus of the p49/STRAP proteins of mammals and vertebrates; and a BUD22 domain was found at the C-terminus of the p49/STRAP proteins and the yeast cellular morphogenesis protein (also known as BUD22). When p49/STRAP gene was overexpressed in cultured cells and in transgenic mice, morphological alterations were observed, both at the cellular and whole organ, structural level.

## Results

### Bioinformatics analysis of p49/STRAP protein

**Figure 1:**
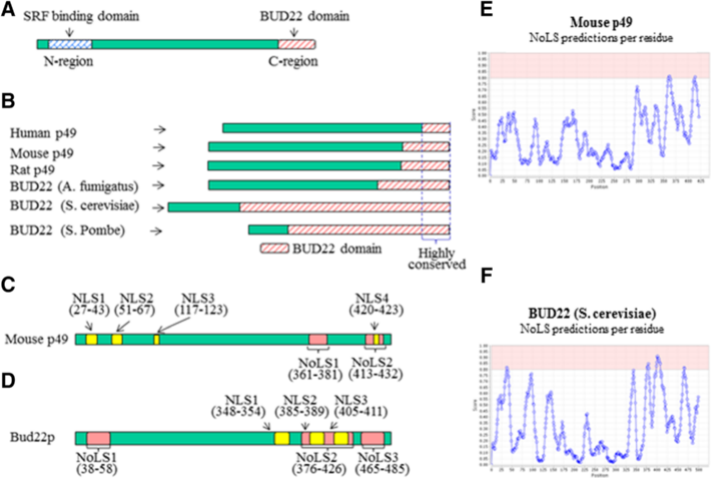
**Bioinformatic analysis of p49 protein sequence. A**. A schematic of the p49 protein with two conserved regions. The SRF-binding domain is at the N-terminus; a BUD22 domain is located at the C-terminus. **B**. A BUD22 domain was found at the C-terminus of human, mouse and rat p49 proteins and three yeast BUD22 proteins, based on results from the Conserved Domain Database (NCBI), Pfam and InterPro databases. The lengths of the BUD22 domains in the six protein sequences illustrated in this diagram were based on the data from the Conserved Domain Database. According to the Conserved Domain Database, the BUD22 domain in human p49 was 29 amino acids in length. We found that this 29 amino-acid region (between two dash lines) was highly conserved in all six protein sequences. **C**. A schematic of the mouse p49 protein (Unipot ID Q9CZ91) which has four nuclear localization signal sequences (NLSs), and two nucleolar localization signal sequences (NoLSs). **D**. A schematic of the BUD22 protein of Baker’s yeast (Unipot ID Q04347), which has 3 NLSs and 3 NoLSs. **E**. A diagram of computational prediction of nucleolar localization signal sequence in mouse p49 protein (Unipot ID: Q9CZ91) using “NOD” (Nucleolar localization sequence Detector) website [44]. **F**. A diagram of computational prediction of nucleolar localization signal sequence in BUD22 of Baker’s yeast (Unipot ID Q04347) using “NOD” (Nucleolar localization sequence Detector) website [44].

The p49/STRAP protein sequences of human, mouse and rat were analyzed using bioinformatics tools. Sequence alignment revealed that there was approximately 60% homology among human, mouse and rat p49/STRAP proteins. Further sequence alignment of 13 mammalian p49/STRAP proteins, including that of chimpanzee and dog, showed a 52% homology in p49/STRAP proteins. Two conserved regions were found at the N-terminus (N-region) and C-terminus (C-region) (Figure 1A, B and Additional file 1: Figure S1). The conserved “N-region” overlaps with the “SRF-binding domain” that was found to interact with SRF in our previous study [1].

The p49/STRAP protein sequences were compared against the “Conserved Domain Database” at the National Center for Biotechnology Information (NCBI), the “Pfam” and the InterPro databases. The C-region of p49/STRAP matched an existing conserved domain in all three databases, specifically the BUD22 domain of the cellular morphogenesis protein that regulates yeast budding selection, polarity and RNA biogenesis (Figure 1B) [10-12]. Each database showed a slightly different length of BUD22 domain in p49/STRAP protein. As for the human p49/STRAP (UniProtKB/Swiss-Prot ID: Q8NEF9), the BUD22 domain was at amino acid 399–428 in the NCBI Conserved Domain database; amino acid 329–428 in the Pfam database, and amino acid 360–428 in the InterPro database, respectively.

Alignment of the BUD22 sequence of 8 yeast strands revealed that there was a conserved domain at the C-terminus (C-region), ranging from amino acid 375 to the end of the sequences (Additional file 1: Figure S2). When the p49/STRAP protein sequences of 13 mammalian species were compared with the 8 yeast BUD22 proteins, it was found that only part of sequences at the end of C-region, approximately 29 amino acids, was homologous among the 21 sequences (Additional file 1: Figure S3), indicating that those sequences represented a highly conserved region in the BUD22 domain (Figure 1B and Additional file 1: Figure S3).

Bioinformatics analysis indicated that there are four NLS sequences in the human, mouse and rat p49/STRAP proteins, while three NLS sequences were found within BUD22 of S. cerevisiae and four NLSs in BUD22 of S. pombe. It was also predicted that mouse p49/STRAP had two nucleolar localization sequences (NoLS), and Baker’s yeast BUD22 had three nucleolar localization sequences (Figure 1C). In addition, it was also predicted that both p49/STRAP and BUD22 proteins would be present in the cytoplasmic compartments, including mitochondria, endoplasmic reticulum, plasma membrane and vesicles of the secretory system (Table 1).

**Table 1:** Bioinformatic analysis of three p49/STRAP proteins and two BUD22 proteins

	**hs. p49**	**mm p49**	**Rat p49**	**BUD22 (B. Yeast)**	**BUD22 (S. Pombe)**
Protein length	429 aa	441 aa	442	519 aa	388 aa
BUD22 domain	29 aa	76 aa	82aa	411 aa	362aa
% (BUD22)	6.80%	17.20%	18.50%	85%	93%
NLS	4	4	4	3	4
ER membrane retention signal	None	None	None	1	1
PTS2	1	None	None	1	none
k-NN prediction	65.2% nuclear	69.6% nuclear	69.6% nuclear	73.9% nuclear	69.6% nuclear
	21.7% cytoplasmic	4.3% cytoplasmic	4.3% cytoplasmic	26.1% cytoplasmic	13% cytoplasmic
	4.3% ER	13% cytoskeletal	13% cytoskeletal		8.7% cytoskeletal
	4.3% vesicles of secretory system	4.3% vesicles of secretory system	4.3% vesicles of secretory system		4.3% endoplasmic reticulum
	4.3% mitochondrial	4.3% plasma membrane	4.3% mitochondrial		4.3% plasma membrane
		4.3% Golgi	4.3% Golgi		

Both p49 and BUD22 proteins were predicted to be present in the nucleus, but also in the cytoplasmic compartments, including mitochondria, endoplasmic reticulum, plasma membrane and vesicles of the secretory system.

### Intracellular distribution of p49/STRAP

**Figure 2:**
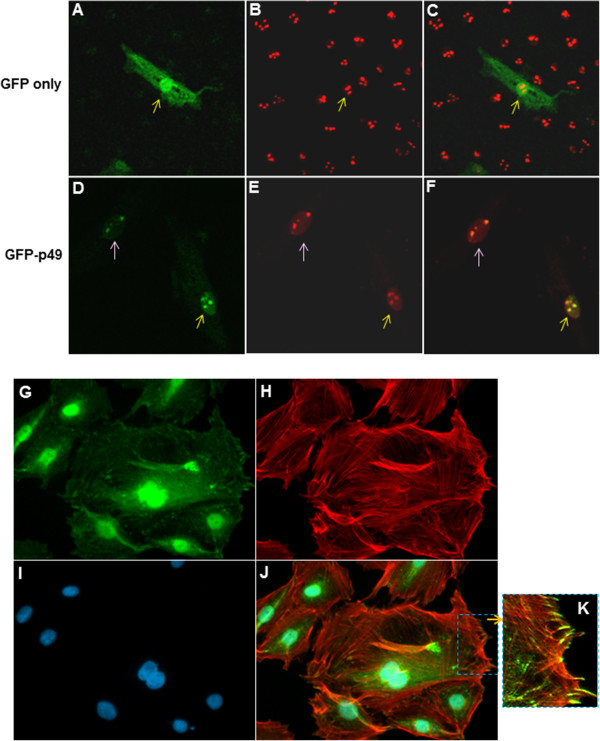
**Intracellular distribution of p49/STRAP proteins. A**-**F**: confocal microscopy revealed that p49 protein co-localized with nucleolin within the nucleolus in H9C2 cells. **A**. GFP protein is distributed in both cytoplasm and nucleus in GFP control plasmid transfected cells. **B**. Anti-nucleolin antibody was used to stain the nucleolus. Red fluorescence showed the nucleolus. **C**. Overlap of images of **A** and **B**. **D**. Transfected GFP-p49 protein was mainly distributed in nucleus and concentrated as dots within the nucleus. **E**. Anti-nucleolin antibody was used to stain nucleolin which is biomarker of nucleolus. Red fluorescence showed the nucleolin were stained as red dots. **F**. Overlap of images **D** and **E**, revealing GFP-p49 protein (in green) and nucleolin (in red) co-localized as yellow dots in the nucleolus. **G**-**K**: The distribution of endogenous p49 protein in H9C2 cells. The figures here revealed the abundance and the distribution of endogenous p49 protein in cells under normal culture condition. **G**. The endogenous p49 protein is widely distributed in the cell, but concentrated in the nucleus. **H**. Actin fiber was stained with rhodamine-phalloidin. **I**. Nuclei were stained with DAPI. **J**. Overlap of images **A**, **B** and **C**, which indicated that the p49 protein was in close proximity to the actin fiber. **K**. Digitally enhanced image showing that the p49 protein appeared to form a “tail” that was co-located at each end of the actin fibers, close to their attachment sites to the inner wall of the cell membrane.

We previously observed that transfected GFP-p49/STRAP fusion protein was mainly localized in the nucleus of NIH3T3 cells, where it acts as a SRF cofactor to regulate SRF-target genes [1]. Indeed, bioinformatics analysis revealed that the mouse p49/STRAP had both nuclear localization signal sequences and nucleolar localization signal sequences (Figure 1C, E).To confirm the bioinformatics result, H9C2 cells were transfected with the GFP-expression control plasmid and GFP-p49/STRAP expression plasmid, respectively. As shown in Figure 2A and C, the green GFP fluorescence was present in the entire cell in GFP-control plasmid transfected cell. By contrast, in the GFP-p49/STRAP expression plasmid transfected cells, the GFP-p49/STRAP protein appeared to be present mainly in nucleus, with a few very bright dots within the nucleus (Figure 2D). We used nucleolin (a biomarker of nucleolus) to co-stain the transfected cells. As shown in Figure 2E, nucleolin was stained in red. When Figure 2D and E were merged (Figure 2F), the bright green-colored GFP-p49/STRAP dots co-localized with the red-colored nucleolin (shown as yellow dots in merged image), indicating that the p49/STRAP protein does localize within the nucleolus.

The p49/STRAP protein is known to be highly expressed in multiple tissues based on Western blotting [1]. To better understand the intracellular distribution of the endogenous p49/STRAP protein, an affinity-purified antibody against a p49/Strap epitope was used for immunochemistry staining of H9C2 cells. As shown in Figure 2G, the endogenous p49/STRAP protein was present in both the nucleus and cytoplasm. In addition, the p49/STRAP protein was widely distributed within the cell in close proximity to the actin fibers (Figure 2J); some of the p49/STRAP proteins appeared to form a “tail” that was co-located at the end of the actin fibers, close to their attachment sites to the inner wall of the cell membrane (Figure 2J and K). These findings suggest that p49/STRAP may play a role in cellular cytoskeletal structure and function via its presence in the cytoplasm.

### p49/STRAP overexpression affects cell morphology

**Figure 3:**
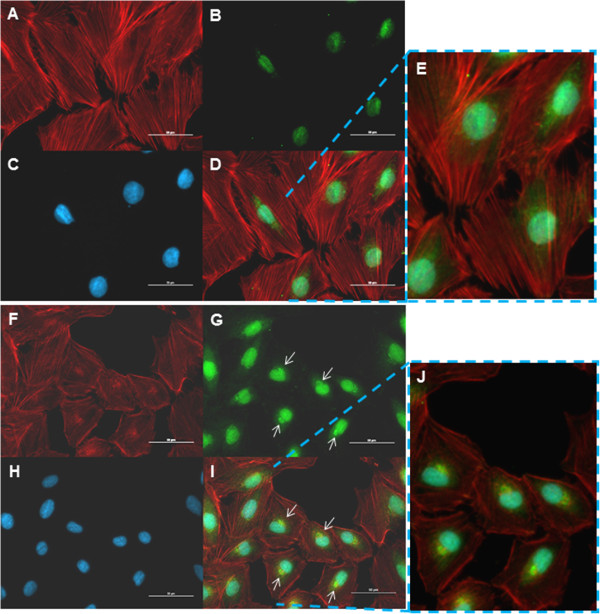
**p49-adenovirus treatment reduced actin contents, and altered actin fiber structure as well as cell morphology. A**-**E**: H9C2 cells that were infected with GFP-control adenovirus. **A**. Actin fibers were well expressed and well organized in control adenovirus infected cells. **B**. The green fluorescence revealed the nuclei which were stained with p49 antibody and an Alexa Fluor 488 labelled secondary antibody. **C**. Nuclei were stained with DAPI. **D**. Overlap of the image **A**, **B** and **C**. **E**. Digitally enhanced image showing control virus infection did not change actin contents and structure in H9C2 cells. There is fainter, less intensive visualization of the GFP protein in the cytoplasm surrounding the nucleus (Figure 3B, D and E). **F**-**J**: H9C2 cells that were infected with p49-adenovirus. **F**. Actin fiber signal intensity was reduced and actin fibers were disorganized. **G**. The green fluorescence revealed both endogenous and adenovirus delivered exogenous p49 proteins that were stained with p49 antibody and an Alexa Fluor 488 labelled secondary antibody. The p49/STRAP proteins were mainly localized in the nuclei. Part of the p49 protein was asymmetrically distributed within the cells (arrows). **H**. Nuclei were stained with DAPI. **I**. Overlap of the images F, G and H. The arrows pointed to the yellow “dots”, which were a result of the merger of green and red fluorescence, which indicated that part of the p49 proteins (in green) were co-localized with actin fiber (in red) within the cells. **J**. Digitally enhanced image showing that p49-adenovirus treatment reduced actin contents, and altered actin fiber structure as well as cell morphology.

**Figure 4:**
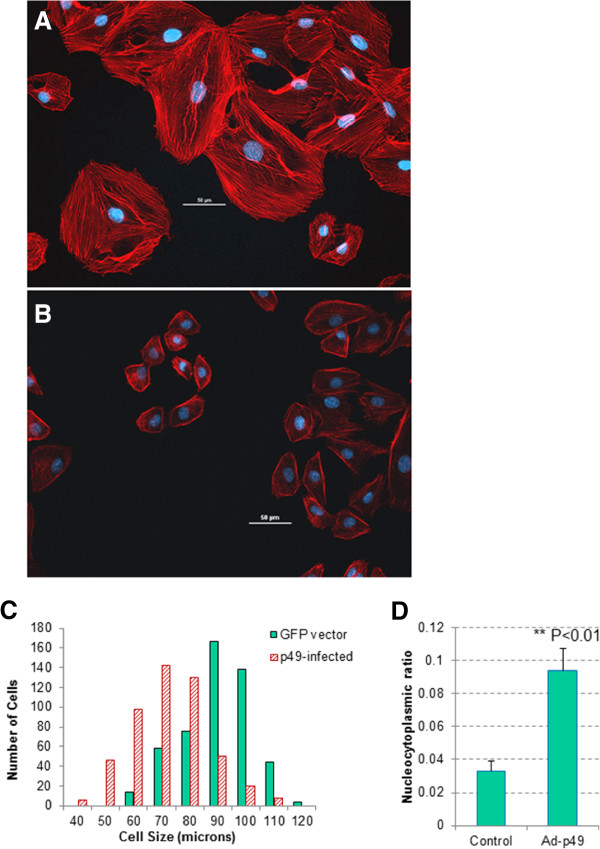
**p49-adenovirus treatment reduced the actin fiber structure and intensity, and changed the cell morphology in term of cytoskeletal structure, cell size, cell shape and nucleocytoplasmic ratio. A**. Control H9C2 cells were infected with GFP-control adenovirus and stained with Phalloidin and DAPI for visualization of F-actin and nuclei, respectively. Control cells showed relatively uniform and normal morphology for H9C2 cell line. Nuclei occupied a small, centrally located region of the cytoplasm. Actin fibers were dense and showed uniform network throughout the cytoplasm of the cell. **B**. P49/STRAP adenovirus infected H9C2 cells were stained with Phalloidin and DAPI for visualization of F-actin and nuclei, respectively. Overexpression of p49/STRAP in H9C2 cells showed smaller overall cell size, along with less uniform cellular shape compared to that of the control. Overall expression of actin was decreased, with significantly less uniformity among visualized fibers. Nuclei of p49/STRAP overexpressed cells were observed to occupy a much greater percentage of the cytoplasm and peripheral location in the cell. **C**. Histogram of cell size and numbers in H9C2 cell samples that were infected with p49-adenovirus versus control adenovirus. The cell size and number were measured under microscope and multiple fields were examined. The overall cell size in p49 adenovirus-infected samples was smaller versus control adenovirus-infected cells (n = 500, p <0.05). **D**. Histogram showed the nuclear to cytoplasmic ratio among control H9C2 cells infected with GFP only and adenovirus p49/STRAP infected cells. Data indicated that overexpression of p49/STRAP resulted in a significant increase in the nuclear to cytoplasmic ratio compared to that of the control (p < 0.05, n = 50). These results suggest that treatment with increased levels of p49/STRAP reduced cytoplasmic volume and overall cellular morphology of H9C2 cells.

To determine the effect of p49/STRAP overexpression on cellular morphology, we infected H9C2 cells with the GFP-control adenovirus or recombinant p49/STRAP adenovirus [13]. At 24 hours post-infection, the cells were fixed and immunocytochemistry staining was performed. The phalloidin was used to stain F-actin. The p49/STRAP antibody and Alexa Fluor 488 labelled secondary antibody were used to stain the cells to visualize the fluorescent intensity of both the endogenous and the exogenous p49/STRAP proteins in the p49/STRAP-virus treated cells versus that of the endogenous p49/STRAP protein only in the control cells infected with GFP-control virus.As shown in Figure 3A-D, in the GFP-control virus infected cells, the actin fibers were present as thick, well aligned bundles (3A, 3D and 3E), and the p49 protein was mainly localized in the nucleus (Figure 3B, D); however, in the p49/STRAP virus treated cells, the structure of the actin fibers was more diffuse, especially in the perinuclear region, and the intensity of the actin fibers was weaker (Figure 3F, I and J). Asymmetric distribution of the p49/STRAP protein was observed within the p49/STRAP treated cells compared to that in the control cells (Figure 3F, H and J). Cell size measurements revealed that H9C2 cells treated with the p49/STRAP adenovirus were smaller (72.3 ± 0.946 SD microns) compared to that of the control adenovirus infected cells (88.1 ± 1.41 SD microns, Figure 4C, p < 0.05, n = 500). In addition, p49/STRAP treated cells showed much larger nucleocytoplasmic ratios compared to that of the control, 1:10.7 ± 0.8 versus 1:30.2 ± 0.5, respectively (p < 0.01, n = 50; Figure 4A, B and D). The overall microscopic observation revealed that increased p49/STRAP protein reduced the F-actin structure and intensity, and changed the cell morphology in term of cytoskeletal structure, cell size, cell shape and nucleocytoplasmic ratio.

### p49/Strap modulated SRF gene expression

**Figure 5:**
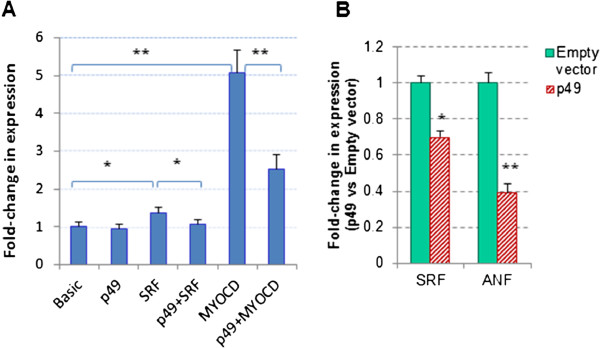
**p49/STRAP repressed SRF and ANF gene expression. A**. Effect of p49 expression on promoter activity of SRF gene. SRF gene promoter has two classic CArG box, therefore, SRF gene itself is regulated by SRF and SRF-cofactors. P49 had repressive effect on SRF gene promoter activity. **B**. p49 repressed the mRNA expression of SRF and ANF genes. * refers to p < 0.05, n = 3; ** refers to p < 0.01, n = 3.

P49/STRAP is a SRF cofactor that co-regulates SRF-target genes [1]. Since there are two SRF-binding sites (the classic CArG boxes) in the SRF gene promoter, the SRF gene itself is also a SRF-target gene subject to the regulation by SRF [14]. To test the hypothesis that p49/STRAP may regulate the SRF gene, a plasmid containing firefly luciferase driven by the SRF gene promoter was used in the luciferase-based promoter activity assay. As shown in Figure 5A, the SRF plasmid transfection induced SRF gene promoter activity (p < 0.05, n = 3); when p49/STRAP was co-transfected with SRF, p49/STRAP repressed the SRF-induced promoter activity (p < 0.05, n = 3). Another SRF cofactor, myocardin was also used in the transfection assay; myocardin overexpression induced SRF gene promoter activity by approximately 5-fold (P < 0.01, n = 3), while p49/STRAP repressed the SRF-promoter activity that was induced by myocardin plasmid transfection (P < 0.01, n = 3). The mRNA levels of SRF and atrial natriuretic peptide (ANF) genes were also measured in response to p49/STRAP overexpression. As shown in Figure 5B, p49/STRAP repressed the expression of both the SRF gene (p < 0.05, n = 3) and the ANF gene (p < 0.01, n = 3).

### Generation of p49/STRAP transgenic mouse lines

The p49/STRAP mRNA/protein is well expressed in multiple tissues, including brain, heart, skeletal muscle, kidney, liver and testis [1]. To test the hypothesis that increased expression of p49/STRAP in vivo may alter the expression of SRF-target genes and affect the morphology of mouse cells and tissues, we generated transgenic mouse lines containing a DNA construct with p49/STRAP cDNA under the transcriptional control of the CMV promoter.

After multiple microinjections and screenings, three transgenic (p49Tg) founder mice were identified. The founder mice were bred to non-transgenic (non-Tg) mice, and three p49Tg lines were thus established. Both Southern blotting and quantitative PCR analysis were used for the detection of the transgene copy number of the three p49Tg lines. Each line was found to have only one transgene copy.

### Death of some p49/STRAP transgenic newborn mice

**Figure 6:**
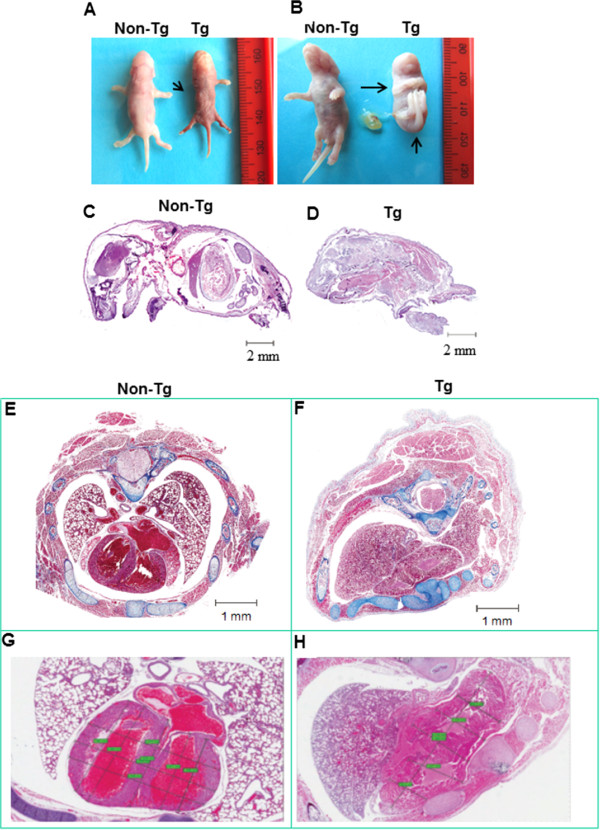
**Morphological change in p49Tg mice versus non-Tg mice. A**. Image of p49Tg newborn (Tg) versus Non-Tg newborn. The arrow indicates the p49Tg newborn missed a leg. **B**. The arrows highlight the contracture deformities of the limbs with additional webbing or non-delineated appendages in the p49Tg newborn. The tissue sections from **C** to **H** were stained with H&E staining. **C** and **D**. Longitudinal section of p49Tg newborn versus non-Tg newborn, which revealed that the spine in the M-p49Tg newborn was shifted around to the abdominal cavity from the back to the front (dorsal to ventral). **E**. Cross sections of non-Tg newborn. **F**. Cross section of Tg newborn with asymmetric thoracic cavity. Both the left and right lungs were unexpanded in the dead newborn the Tg mouse. **G**. Tissue section of non-Tg heart. **H**. Tissue section of p49Tg heart, which revealed malformed heart.

The p49Tg founder mice that survived to adulthood were fertile without apparent abnormality in reproduction. The p49Tg offspring had their genotype confirmed by PCR. An examination of 82 litters from 6 generations of the p49Tg mice revealed that the transgenic offspring separated into two groups based on p49/STRAP mRNA expression level and their phenotype (either malformed or normal-appearing). A close check of three mouse generations revealed that among 137 newborn p49Tg mice, 60 newborns were malformed, which accounted for 43% of the p49Tg offspring. The malformed group of newborn P49Tg mice (M-p49Tg) displayed serious defects and almost all of them were either dead at birth or died immediately thereafter (Figure 6A, B). These malformed mice had elevated level of p49/STRAP mRNA expression (by real-time RT-PCR). The other group of newborn P49Tg mice were grossly normal-appearing (NA-p49Tg), had no apparent anatomic defects and most of these P49Tg mice survived without visible abnormality. These NA-p49Tg mice had similar levels of p49/STRAP mRNA expression compared with that of the non-Tg mice (by real-time RT-PCR). However, their p49/STRAP transgenic status was confirmed by Southern blot analysis and/or by TaqMan copy number assay.Longitudinal sections of the dead newborn mice revealed that the spine in the M-p49Tg newborn was shifted around to the abdominal cavity from the back to the front (dorsal to ventral, Figure 6C and D). Cross sections showed that the thoracic cavity was asymmetric and the left side of the chest was smaller than that of the right (Figure 6E and F). Both the left and right lungs were unexpanded in the M-p49Tg mice that were dead at birth, and the heart morphology was substantially abnormal, without an appropriate division and/or formation of separate cardiac chambers in the M-p49Tg mice vs. non-Tg (Figure 6G and H).

**Figure 7:**
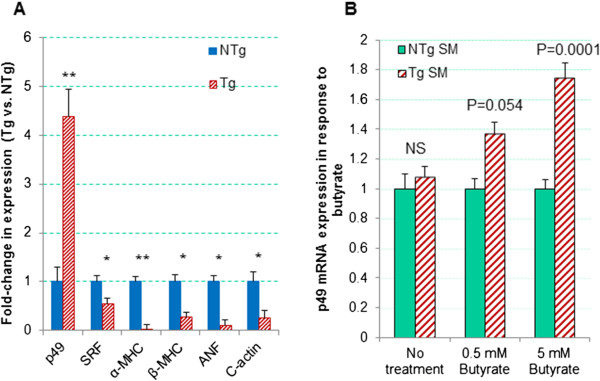
**Gene expression of p49 and other genes in p49Tg mouse tissue and cells versus that of non-transgenic mice. A**. Gene expression in malformed p49Tg newborns versus NTg newborns. The data indicated that p49 overexpression altered the expression of cytoskeletal genes. * refers to p < 0.05, n = 3; ** refers to p < 0.01, n = 3. **B**. Aortic smooth muscle cells (isolated from 3 month old Tg and NTg) were treated with sodium butyrate for 24 hours. P49 expression was not significant between Tg cells and NTg cells in the absence of sodium butyrate (No treatment). There was slight increase in p49 expression Tg cells in the presence of 0.5 mM sodium butyrate, but not significant (p = 0.054, n = 3). p49 mRNA was significantly increased in the presence of 5 mM sodium butyrate (p < 0.01, n = 3).

In the malformed M-p49Tg mice that were dead at birth, the p49/STRAP expression was increased, while SRF, ANF, cardiac actin, α-MHC and β-MHC were decreased, in the whole body preparation, indicating that p49/STRAP overexpression altered the expression of these cardiac genes that are important in cardiac development and cellular cytoskeletal integrity (Figure 7A) the CMV promoter was used to drive the expression of p49/STRAP transgene expression, the possibility of CMV promoter silencing was considered to be the possible mechanism in the normal-appearing mice without elevated p49/STRAP expression. A number of reports indicate that CMV silencing can be reactivated by sodium butyrate [15,16]. Therefore, we sought to test this possibility in aortic smooth muscle (ASM) cells that were isolated from 3 month old Tg and NTg mice. P49/STRAP mRNA expression was measured in response to sodium butyrate treatment. As shown in Figure 7B, p49/STRAP mRNA level was slightly increased after the Tg ASM cells were treated for 24 hours in the presence of 0.5 mM sodium butyrate (p = 0.054, n = 3). However, p49/STRAP mRNA level was increased significantly after the Tg ASM cells were treated for 24 hours in the presence of 5 mM sodium butyrate (p < 0.01, n = 3). These findings indicate that in the present study, CMV promoter silencing may well have repressed p49/STRAP expression in those NA-p49Tg group, which were without abnormal phenotype and without increased p49/STRAP gene expression.

## Discussion

In the present study, we found that the p49/STRAP protein has a SRF-binding domain at the N-terminus and a BUD22 domain at the C-terminus. The N-terminal domain containing the SRF-binding domain was highly conserved among mammalian species, xenopus and zebrafish. The C-terminal domain containing the BUD22 domain was conserved not only among mammalian p49/STRAP proteins, but also conserved among yeast cellular morphogenesis proteins that regulate cell morphology and polarity. In addition, overexpression of p49/STRAP in vitro and in vivo indeed changed the cell actin content, cell size and cell polarity.

P49/STRAP is one of the SRF-binding proteins/cofactors that participate in the regulation of cytoskeletal and muscle-specific genes [1]. The SRF protein is a major transcription factor in regulating cardiac, skeletal and smooth muscle genes [17]. SRF has a DNA binding domain at the N-terminus that binds to the SRE (CArG box) DNA sequence, and a transcriptional activation domain at the C-terminus that is required for serum induction [18]. SRF and its cofactors usually form a protein complex which binds to SRF target gene promoters and consequently activates or represses the target genes [1]. Therefore, SRF cofactors play an important role in assisting SRF to conduct tissue-specific and development stage-specific regulation of its target genes [1]. Among SRF cofactors, the TCF cofactor, myocardin and MRTFs bind to the SRF N-terminus, while p49/STRAP protein has been shown to bind to the SRF transcriptional activation domain [1,5,17]. It has been shown that the p49/STRAP protein is usually located within a protein complex containing the SRF and myocardin proteins. Therefore, p49/STRAP is able to modulate the SRF transcriptional activation [1].

The localization of p49/STRAP protein has been studied experimentally, either through transfection assays or proteomic analyses [1,13,19-21]. For instance, Lamond’s group has reported that p49/STRAP (SRFBP1) (ID # Q96DK2, or IPI00304493) is among a number of proteins, including Werner syndrome helicase, Bloom’s syndrome helicase and nucleolin, that are localized in the nucleolus [20,21]. Recently, Tafforeau et al. demonstrated that p49/STRAP (SRFBP1) is in the nucleolus and serves as a pre-rRNA processing factor which regulates ribosome biogenesis [22]. The authors stated that p49/STRAP did not have a yeast homologue [22]. However, we have now observed that p49/STRAP does in fact, have a yeast homologue, namely BUD22 based on the domain search in NCBI, Pfam and InterPro databases. In addition, we observed that p49/STRAP is localized in both the nucleus and the nucleolus, which is similar to what has been reported for BUD22. BUD22 is one of the proteins that regulate yeast bud site selection and polarized cell growth in response to both external and internal stimuli [23]. Polarized cell growth is a phenomenon that occurs in virtually all cells and it is essential for many important cellular events such as multicellular organ development and cell movement.

In addition, cell size and morphology are impacted by ribosome biogenesis which affects growth rate and cell size [24,25]. Cell morphology is also regulated by various BUD proteins. It has been shown that BUD22 is involved in ribosome biogenesis, which affects both cell growth rate and cell size [10,12]. Therefore, it is likely that p49/STRAP may regulate cell size and morphology through its partnership with SRF in regulating cytoskeletal genes, and may also regulate cell morphology and polarity with its BUD22 domain.

Cytoskeletal and muscle-specific genes are key components of cell morphology. Many of these genes are regulated by SRF and its cofactors. In the present study, p49/STRAP has been found to reduce actin fiber content and change cell morphology in cultured cells. Interestingly, the effect of p49/STRAP transfection on reducing cellular actin correlated with reduced actin expression in the malformed p49Tg newborns. The widespread structural and especially cardiac abnormalities in the dead newborns could be the consequence of altered expression of important cytoskeletal genes such as actin and other cardiac muscle-specific genes which include α-MHC and β-MHC, ANF and SRF itself. The p49/STRAP protein, as a co-factor of SRF, might be involved in the regulation of cytoskeletal and muscle-specific genes through potentially two different but related mechanisms. First, p49/STRAP may interact with SRF protein in the promoter of various cytoskeletal and muscle-specific genes and modulate their expression; second, p49/STRAP may indirectly modulate cytoskeletal and muscle-specific genes via regulating the expression of the SRF gene itself, which is a major regulator of many other SRF-target genes [14,26]. The altered cellular actin dynamics may also indirectly regulate SRF activation [27-29].

In the transgenic mouse lines which were created in the present study, the p49/STRAP transgene expression was under the control of a CMV promoter, which is widely used to drive the ubiquitous expression of a transgene. Since the transgene overexpression can sometimes cause artifacts such as transcriptional squelching and may change balanced gene dosage and affect downstream regulation, it is also possible that the experimental results may not reflect the real function of a transgene. This could happen when the overexpression of a transgene is very high [30,31]. However, in the present study, all three p49Tg lines each has only one transgene copy, and none of them had high transgene expression level. Therefore, the phenotypes observed in the present study are most likely to be the result of p49/STRAP overexpression instead of artifacts.

The p49Tg mice appeared to separate into two different groups, those with severe defects that died at birth, and those without apparent defects/abnormality at birth and continued to live in the absence of noticeable phenotype. The main difference between these two groups of p49Tg offspring in terms of gene expression was in the level of p49/STRAP transgene expression: the p49Tg with anatomical defects had elevated p49/STRAP gene expression, while the p49Tg mice without defects had no significant elevation in p49/STRAP expression and their levels were similar to that of non-Tg littermates. These findings suggest that p49/STRAP overexpression clearly plays a critical role in the manifestation of the abnormal phenotype observed in the p49Tg mice. The finding that sodium butyrate-induced reactivation of p49/STRAP transgene mRNA expression occurred in isolated aortic smooth muscle cells of the NA-p49Tg mice supports the notion that CMV promoter silencing most likely contributed to the repression of p49Tg transgene expression in the subset of the p49Tg mice that were without elevated p49/STRAP expression and without abnormal phenotype [32,33].

The CMV promoter has been reported to have one CpG island with 14 CpG sites, which are subjected to DNA methylation or hypermethylation. Consequently, the CMV promoter is sometimes repressed by DNA methylation, which results in the decline, or complete silencing of the transgene expression [15,16,32,33]. The CMV promoter that was used in the present p49Tg study is 654 bp long, which is part of the pcDNA3-p49/STRAP expression plasmid [1]. The web-based software “CPGPLOT” was used to scan the sequence [34], and a CpG island was found in the CMV promoter that could potentially be methylated (figure not shown), which may have silenced the p49/STRAP transgene in some mice. CMV promoter methylation could occur both in vitro and in vivo [35]. The degree of repression may vary from mouse to mouse, and may even be different among organs within a given animal [36]. Furthermore, the degree of CMV promoter repression could be related to the length of the promoter and the GC content in the CMV promoter sequence which is used in the given DNA construct; it is also related to the transgene integration site [36].

In addition to DNA methylation, which may have occurred in the CMV promoter, it is known that histone deacetylation may also affect the CMV promoter activation [33]. Previously, we observed that overexpression of p49/STRAP itself altered the intracellular level of NAD, reduced the NAD/NADH ratio, and also induced the deacetylation of the SRF protein [13]. It is therefore plausible that overexpression of p49/STRAP itself could also change the acetylation status of other proteins, such as histones, and/or cause other epigenetic changes, including CMV promoter silencing. Epigenetic stability is important for the oocyte-to-embryo transition. Disruption of epigenetic stability could result in embryos displaying a large variety of phenotypes and growth defects during mouse embryogenesis [37]; perhaps similar to what was observed in some of the malformed p49Tg mice with elevated p49/STRAP expression. Similar to the reversal of other epigenetic modifications, DNA methylation at the CMV promoter could also be reversed by means of pharmacological intervention. It is plausible that reversing CMV methylation could potentially reactivate the transgene expression [36]. Future studies to test these possibilities will be of interest.

## Conclusions

Taken together, our findings indicate that p49/STRAP participates in the regulation of cytoskeletal gene expression and in the regulation of cell size as well as morphology/polarity. Elevated expression of p49/STRAP in P49Tg mice resulted in various organ defects, including asymmetric (or polarized) distribution of the vertebral spine and organs with altered expression of cardiac and skeletal muscle genes. Inasmuch as p49/STRAP is a SRF binding protein, and we have observed that it is also a homolog of the yeast BUD22 protein (which is known to regulate cell size and polarity), it is plausible that p49/STRAP may serve as a bridge that links two important pathways: the SRF-related pathway in transcriptional regulation of cytoskeletal genes and cell size (SRF mediates PTEN’s regulation of cell size), and the BUD22-related pathway that regulates cell size, cell morphology and polarity. We propose that the increased expression of p49/STRAP that is observed in aged human and rodent cells may mediate some of the observed changes in cellular morphology and the appearance of some of the markers of cellular senescence, including changes in actin structure and other intracellular-cytoskeletal proteins. Future studies in this area will likely yield substantial information.

## Methods

### Bioinformatics analysis

The p49/STRAP sequences were analyzed using web-based and/or freely-downloaded bioinformatics tools, including “Conserved Domains” Blast [38], Pfam [39], SMART [40,41], ExPASy [42], InterPro [43], NOD (Nucleolar localization sequence Detector) [44], PSORT II [45], iPSORT [46], and MultAlin [47]. The CMV promoter sequence was analyzed using EMBOSS CpGPlot software [34].

### Immunocytofluorescence

For visualization of intracellular p49/STRAP and actin fibers, H9C2 cells were equally seeded onto six-well plates and grown in DMEM supplemented with 10% FBS at 37°C and 5% CO2 for 24 h. Upon attachment and ~80% confluence, H9C2 cells were infected with control adenovirus and recombinant p49/STRAP adenovirus for 24 h and 48 h, respectively. The cells were then fixed in 3.7% formaldehyde for 10 min at room temperature. The fixative was removed by washing with PBS and cells were permeabilized in 0.2% Triton X-100 for 5 min. Cells were washed with PBS blocked with 3% BSA for 1 h at room temperature. Samples were then incubated with primary rabbit p49/STRAP antibody (1:200) for 2 h at room temperature. Fluorescent secondary antibody signal was developed using Alexa Fluor 488 goat anti–rabbit (1:500) for 1 h at room temperature. F-actin was stained using rhodamine-phalloidin (1:200) for 20 min. Samples were then counterstained for 5 min with DAPI (1:1000) to label the nuclei. Images were examined with a 40× PLAN Fluor 1.3 objective (Nikon, Inc.) at room temperature using an Eclipse TI inverted fluorescence microscope (Nikon, Inc.) and captured and analyzed with Photometrics CoolSNAP HQ2 and NIS Elements AR software (v. 4.10.01 Nikon, Inc.). Images were exported as TIFF files.

### Affinity-purified antibody against p49/STRAP protein

As described in our previous study, we generated a p49/STRAP antibody against a peptide (KSKKGTEDALLKNQRRAQ) of the p49/STRAP protein, which showed high specificity to the p49/STRAP protein [1]. In the present study, the polyclonal antibody was commercially purified (Genemed Synthesis Inc.) using affinity chromatography with the same peptide that was used for the generation of the p49/STRAP polyclonal antibody.

### Recombinant adenovirus infection

The p49/STRAP recombinant adenovirus was generated using the AdEasy system (a generous gift from Dr. Vogelstein, Johns Hopkins Oncology Center, MD) as was previously reported [13].

H9C2 cells were grown in 6-well plates and infected with control adenovirus and recombinant p49/STRAP adenovirus for 24 h and 48 h, respectively. Adenovirus treatment groups were performed in triplicate. After fixation, the cells were viewed using a 20× PLAN Fluor 0.5 objective (Nikon, Inc.) at room temperature, and the images were captured and analyzed. Post-treatment H9C2 cell size was determined using the measurement module in NIS Elements AR software (Nikon, Inc.). Cell size was measured through randomized field of view analysis for each treatment group in triplicate. For each viral infection treatment group (Ad-EGFP and Ad-p49/STRAP), a total of 500 cells were counted and analyzed. The nuclear to cytoplasmic ratio was measured using Nikon eclipse software.

### Luciferase reporter assay

The plasmid DNA constructs used in the current study, including the p49/STRAP, SRF full-length cDNA and myocardin expression vectors, as well as the SRF gene promoter-luciferase vector, have been previously described [1,48].

Transient transfections were performed using Lipofectamine 2000 (Invitrogen, Carlsbad, CA) as previously described [1,9].

### Generation of CMV-p49/STRAP transgenic mouse lines

The plasmid pcDNA3-p49/STRAP containing the mouse p49/STRAP cDNA (accession # AY611629) was constructed as previously described [1]. Briefly, to make a transgenic DNA construct, a 2.7 kb DNA fragment containing CMV promoter, the mouse p49/STRAP cDNA and the BGH polyadenylation signal sequence was released from the plasmid pcDNA3-p49/STRAP using NruI and DraIII restriction enzymes, and was separated on a low melting gel and then isolated and purified as previously described [9,49].

The microinjection of the DNA construct and generation of the transgenic mouse line were conducted at the Genetically Engineered Mouse Models (GEMM) Core of the University of Arizona. Three p49/STRAP transgenic (p49Tg) lines were generated. The TaqMan copy number assay was used for the determination of transgene copy number. The target gene was p49/STRAP (assay ID: Mm00488691) and the reference gene was Tfrc (Life technology). Southern blot analysis was also employed for the determination of the transgene copy number using the method previously described [9]. In all experiments, age and sex-matched non-transgenic littermates were used for comparison with the p49Tg mice. For each time point, there were three independent biological replicates. The studies were conducted with the approval of the Institutional Animal Care and Use Committee (IACUC) at Central Arkansas Veterans Healthcare System (IACUC# 4-02-03) and in accordance with the NIH Guiding Principles for Research Involving Animals and Human Beings.

### Histological analysis

The bodies of stillborn mice and various tissue samples from euthanized newborn or stillborn mice were placed in 10% neutral buffered formalin overnight. After fixation, the samples were subjected to a dehydration series and embedded in paraffin. Sections (3–4 uM) were stained using standard hematoxylin and eosin (HE) or Masson trichrome staining protocols [9].

The histological images of the stained sections were obtained by scanning the microscope slides using a ScanScope Digital Slide Scanner at the magnification 40× (Aperio, Vista, CA).

### Real-time RT-PCR

All RNA samples were isolated from mouse tissues using the miRNeasy Mini kit and RNase-free DNase I (Qiagen) [50,51]. The RT-PCR was carried out using reagents purchased from Applied Biosystems. The real-time RT-PCR was performed using the following primers: 1) p49/STRAP forward, 5′-gaggctttgagagtgtgaagca-3′ and reverse, 5′-cttcccaggacggatgca-3′; 2) SRF forward, 5′-accccaccacagaccagaga-3′ and reverse, 5′-ggtaggtgagatctggctcttca-3′; 3) ANF forward, 5′-tgggacccctccgatagatc-3′ and reverse, 5′-agcgagcagagccctcagt-3′; 4) α-MHC forward, 5′-tggtccacattcttcaggatt-3′and reverse, 5′-cctctaggcgttccttctctg-3′; 5) β-MHC forward, 5′-tcctgctgtttccttacttgc-3′ and reverse, 5′-ctgagccttggattctcaaac-3′; 6) cardiac actin forward, 5′-gctttggtgtgtgacaatgg-3′ and reverse, 5′-ctgtcccatacccaccatga-3′. The 5S RNA reference primers (Exiqon) that were used as endogenous control.

The PCR amplification was performed in a StepOnePlus Real-Time PCR System (Applied Biosystems). Relative expression values were obtained by normalizing CT values of the mRNA genes in comparison with CT values of the endogenous control (5S RNA) using the CT method [50,52].

### Isolation of mouse aortic smooth muscle cells and sodium butyrate treatment

All studies were conducted with IACUC approval. Following euthanasia as per approved protocol, the mouse aortic smooth muscle (ASM) cells were isolated from both normal-appealing p49Tg and their non-Tg littermate mice according to the method described by Ray et al. [53]. Subsequently, the ASM cells were cultured in DMEM medium containing 15% fetal bovine serum, 100U/ml Penicillin, 100 ug/ml Streptomycin and 2.5 ug/ml Fungizone (Invitrogen). For treatment, the ASM cells were cultured in 6-well plates in the presence of sodium butyrate at 0.5 mM and 5 mM final concentration for 24 hours. The ASM cells cultured in the absence of sodium butyrate on the same plates were used as controls. After treatment, the cell in each well was washed with PBS and then subjected to total RNA isolation procedure using the miRNeasy Mini kit and RNase-free DNase I (Qiagen) [50,51].

### Statistical analysis

Data were analyzed by two independent observers, who were blind to the transgenic status of the mice. A two-tailed *T*-test was used to determine the differences between the two groups. Bonferroni correction was applied to multiple comparisons. A *P* value of <0.05 was considered to be statistically significant.

## Abbreviations

P49/STRAP: SRF-Dependent Transcription Regulation-Associated Protein; SRF: Serum response factor; SRFBP1: SRF binding protein 1; BUD22: Bud site selection protein 22; Tg mouse: Transgenic mouse; CMV: Cytomegalovirus; RT-PCR: Reverse transcription polymerase chain reaction.

## Competing interests

The authors declare that they have no competing interests.

## Authors’ contributions

Conceived and designed the experiments: XMZ, JYW. Performed the experiments: XMZ, SCR, SL. Analyzed the data: XMZ, GA, SCR, JYW. Contributed reagents/materials/analysis tools: XMZ, GA, SCR, JYW. Wrote the paper: XMZ, GA, JYW. Participated in the coordination of the study: GA. Overall interpretation of results: JYW. All authors read and approved the final manuscript.

## Supplementary Material

Additional file 1: Figure S1, S2 and S3 Bioinformatics analysis of p49/STRAP protein and search results of sequence databases. Click here for file
