# Supplementary material for: Overexpression of p49/STRAP alters cellular cytoskeletal structure and gross anatomy in mice
Source: BMC Cell Biol. 2014 Sep 2;15:32. doi: 10.1186/1471-2121-15-32 (PMC4160719; doi:10.1186/1471-2121-15-32)
Supplement: Additional file 1: Figure S1, S2 and S3 — Bioinformatics analysis of p49/STRAP protein and search results of sequence databases. [file 1471-2121-15-32-S1.pdf]

Additional file 1

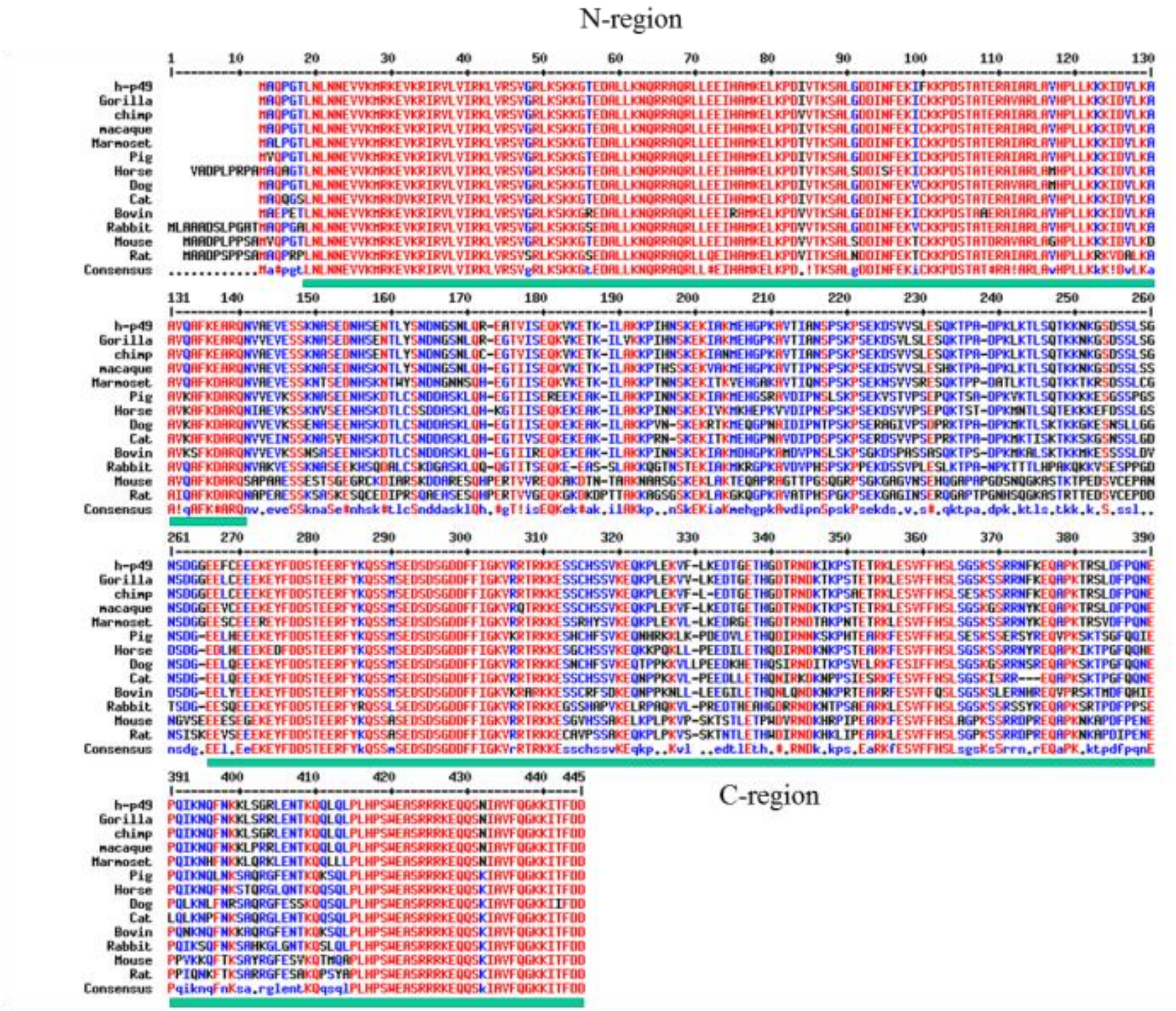

Figure S1.

Figure S1. Alignment of p49 protein sequences in 13 mammalian species. Two conserved regions (N-region and C-region) were revealed.

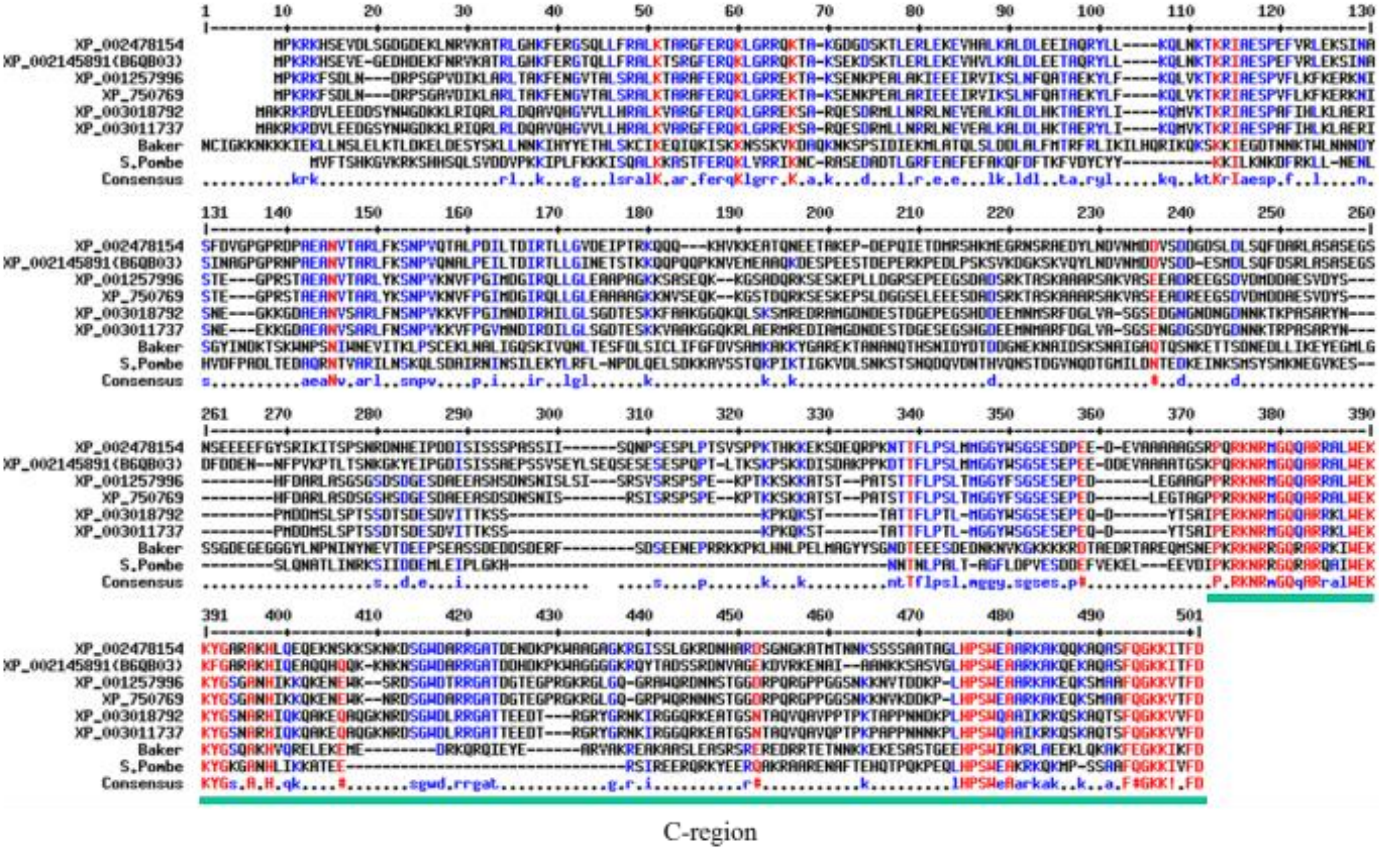

Figure S2.

Figure S2. Alignment of cellular morphogenesis protein (BUD22) of 8 yeast strains. One conserved regions (C-region) was revealed. The first 40 amino acids in Baker's yeast, which does not match other sequences, were not included. These 40amino acids are: MPSESSVSIY KLDQLEYQYH YLTKSLQKFE PRYPKTAKLY.

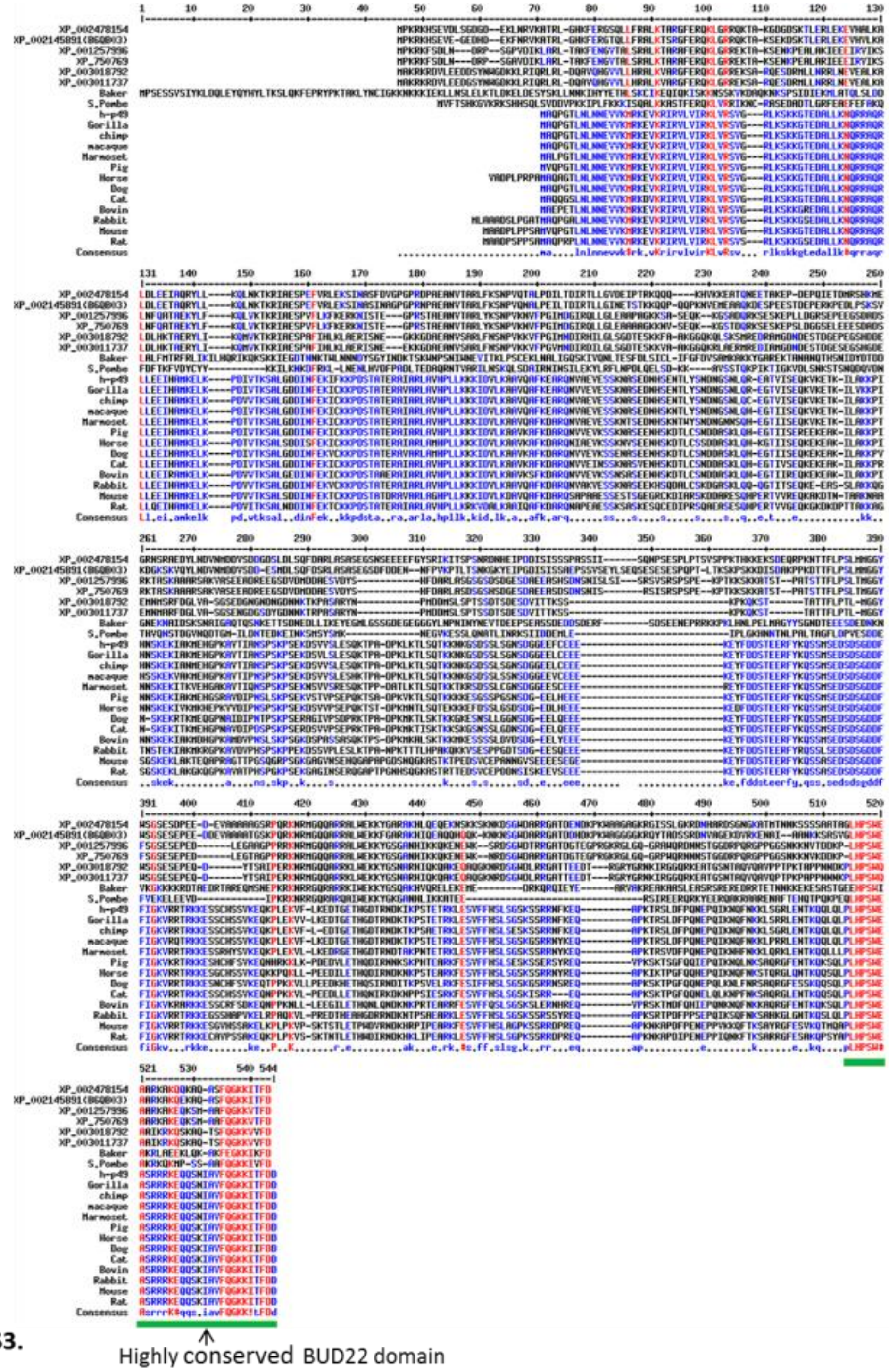

Figure S3.

Figure S3. Alignment of p49 protein sequences of 13 mammals and cellular morphogenesis protein (BUD22) of 8 yeast strains. One conserved regions (C-region) was revealed. The first 40 amino acids in Baker's yeast, which does not match other sequences, were not included. These 40amino acids are: MPSESSVSIY KLDQLEYQYH YLTKSLQKFE PRYPKTAKLY.
